# Editorial: Plant Programmed Cell Death Revisited

**DOI:** 10.3389/fpls.2021.672465

**Published:** 2021-03-25

**Authors:** Joanna Kacprzyk, Arunika H. L. A. N. Gunawardena, Francois Bouteau, Paul F. McCabe

**Affiliations:** ^1^School of Biology and Environmental Science, University College Dublin, Dublin, Ireland; ^2^Biology Department, Dalhousie University, Halifax, NS, Canada; ^3^Laboratoire Interdisciplinaire des Énergies de Demain, Université de Paris, Paris, France

**Keywords:** plant programmed cell death, abiotic stress, plant development, cell death proteases, transcription factors

Plant life cannot exist without programmed cell death (PCD). Both plant developmental processes and responses to environmental factors are modulated by highly controlled, localized cell death events (Kacprzyk et al., 2011; Locato and De Gara, 2018). A detailed understanding of these essential pathways and their regulation is therefore required, especially in the light of challenges imposed on plant health and productivity by an increasingly volatile climate. Reassuringly, this Research Topic gathers contributions underscoring that the plant PCD research is coming out of age and indeed holds the potential to drive the development of novel stress tolerant crop cultivars.

The two review articles of this Topic discuss critical aspects of plant cell death pathway: its transcriptional control (Burke et al.) and the proteases involved in execution of cell death process (Balakireva and Zamyatnin). Burke et al. highlights the role of transcription factors (TFs) in the regulation of plant PCD occurring in response to abiotic and biotic environmental triggers, complementing a recent publication by Cubría-Radío and Nowack (2019), who focused on TFs involvement during developmental PCD. Burke et al. surveyed TFs from NAC, ERF and WKRY families that are involved in life-death decisions in response to environmental perturbations, including those modulating mitochondrial stress signaling. This work provides a starting point for integrative analysis of gene regulatory networks involved in PCD induced by abiotic and biotic stresses, and further elucidation of core transcriptional mechanisms driving the cell death processes in plants. Following PCD activation, proteolytic cascades are important elements of cell death pathways in animals, responsible for signal transduction (initiation caspases) and degradation of cellular components (effector caspases) (Crawford and Wells, 2011; Galluzzi et al., 2018). While canonical caspases are absent in plant genomes, numerous proteases have been shown to play a role in plant PCD, often displaying caspase-like specificity (Salvesen et al., 2016). In this Research Topic, Balakireva and Zamyatnin compare the key characteristics of protease function during cell death in animal and plant cells. The authors propose that proteolytic death-inducing cascades also exist in plant cells, although their participants are different in origin, but similar in function, to those described in animals (see Figure 1 in Balakireva and Zamyatnin). We expect that the application of increasingly accessible proteomics-based approaches will test this hypothesis in the near future.

The Original Research Articles from this topic uncover different aspects of both developmentally regulated and environmentally induced PCD modulation.

Dauphinee et al. used lace plant leaf morphogenesis, a unique model system for studying developmentally regulated PCD, to study the cross talk between cell death and autophagy. Authors demonstrate through the chemical modulation of autophagy, that the process does not have a direct role in the induction of developmental PCD during lace plant leaf morphogenesis and primarily contributes to cell survival. These findings are an important contribution to the ongoing debate on the role of autophagy in plant cell death (Bozhkov, 2018).

The Earth's changing climate is leading to increased frequency of extreme weather events that may lead to severe crop damage. Thankfully, plants have developed diverse strategies to deal with environmental stresses, and PCD is part of this defense repertoire (Kacprzyk et al., 2011; Locato and De Gara, 2018). In this Research Topic, Pegg et al. provide novel insights into PCD-mediated formation of aerenchyma, a tissue comprising empty spaces that facilitates the gas exchange and consequently a key morphological adaptation for waterlogging tolerance (Mustroph, 2018). The study characterized the flooding-induced aerenchyma formation patterns and the associated cell wall carbohydrate modifications within the vascular stele of three Fabacea species (pea, chickpea, and runner bean). The authors identified a localized (and likely carefully controlled) pectin de-methyl-esterification as a putative mechanism paving the way for enzymatic degradation of cell walls and cell death in the aerenchyma forming tissues. Efforts to improve the flooding tolerance in legumes are likely to significantly benefit from this work. The theme of PCD in plant stress tolerance is continued by Chua et al. who explored the effect of the cyanobacteria *Nostoc muscorum* exometabolites on the heat-induced PCD in Arabidopsis root hairs. In their elegantly designed study, the authors demonstrate the suppression of stress-induced plant PCD due to uptake of cyanobacteria-derived proline. The findings suggest a novel mechanism for increased plant stress tolerance when Nostoc is used as a biofertilizer, and may therefore stimulate development of more effective strategies where Nostoc strains with increased proline secretion are generated. The rates of PCD in root hairs were also studied in this Research Topic as a potential marker for the variety stress tolerance (Chua et al.). These authors used the root hair assay (Kacprzyk et al., 2014) to score the PCD rates in barley and wheat seedlings and estimate their basal-, induced- and cross-stress tolerance. The stressed-induced PCD responses were an effective biomarker for preliminary identification of stress tolerant cereal varieties prior to large scale field testing, making the study highly relevant from an agricultural point of view.

Collectively, the contributions from the Research Topic emphasize the progress in the field of PCD research and its potential to deliver solutions from the laboratory to the farm. They also highlight the still unanswered questions about the death of plant cell—and hence the exciting opportunities for further research!

## Author Contributions

All authors listed have made a substantial, direct and intellectual contribution to the work, and approved it for publication.

## Conflict of Interest

The authors declare that the research was conducted in the absence of any commercial or financial relationships that could be construed as a potential conflict of interest.
